# Comparative evaluation of treatment with low-dose aspirin plus dipyridamole versus aspirin only in patients with acute ischaemic stroke

**DOI:** 10.1186/1471-2377-12-67

**Published:** 2012-08-06

**Authors:** Lola Arnarsdottir, Clara Hjalmarsson, Lena Bokemark, Björn Andersson

**Affiliations:** 1 Department of Internal Medicine, The Stroke Unit, Sahlgrenska University Hospital, S-413 45, Göteborg, Sweden

**Keywords:** Pre-stroke, Antiplatelet, Post-stroke, Treatment, Mortality

## Abstract

**Background:**

Previous studies have suggested that pre-stroke treatment with low-dose aspirin (A) could reduce the severity of acute ischaemic stroke, but less is known on the effect of pre-stroke treatment with a combination of aspirin and dipyridamole (A + D) and post-stroke effects of these drugs. The aim of the present study was to evaluate the effect of this drug combination on acute and long-term prognosis of ischaemic stroke.

**Methods:**

Patients without atrial fibrillation admitted to the stroke unit with acute ischaemic stroke (n = 554) or TIA (n = 108) were studied during acute hospital care and up to 12 months after discharge from hospital.

**Results:**

Prior to acute stroke 62 patients were treated with A + D while 247 patients were treated with A only. No beneficial effects of the combination A + D compared to A only were noted on stroke severity and/or acute in-hospital mortality. However, survival analysis by Cox-proportional hazard model demonstrated lower 12-months all-cause mortality in patients discharged with A + D (n = 275) compared with patients on A only (HR, 0.52; CI, 0.32-0.86; p = 0.011; n = 262) after adjusting for age, baseline NIHSS, previous stroke, previous myocardial infarction and type 2 diabetes. We also noted a tendency towards lower all-cause mortality at 3 months with use of A + D, but this was not statistically significant (p = 0.12).

**Conclusions:**

Pre-stroke treatment with a combination of low-dose A + D does not reduce the severity of acute stroke, nor does it reduce the acute in-hospital mortality. However, treatment with A + D at discharge from hospital is seemingly associated with lower long-term mortality compared with A only, contrary to the results from previous randomised studies. However, our results must be interpreted with extreme caution considering the non-randomised study design.

## Background

Several reports have suggested that patients suffering an acute ischaemic stroke while treated with aspirin have less severe strokes than those who are not on aspirin [1-3]. However, other studies have failed to demonstrate such an effect [4-6]. It has been hypothesized that antiplatelet drugs, such as aspirin, may limit the size and extent of thrombosis and subsequent emboli and thereby reduce stroke volume [1].

Dipyridamole (D) is clinically distributed in extended-release form and is primarily recognized as an antithrombotic agent with antiplatelet properties. It has been suggested to reduce the inflammatory process during the sub-acute stroke stages which may be beneficial in the ischaemic stroke setting [7]. This drug has, in combination with aspirin, been shown to improve outcome, reduce stroke recurrence and vascular death in patients with previous stroke [8-13]. There is, however, a paucity of data on the effect of pre-stroke treatment with A + D and the effects of such a combination on early post-stroke outcome.

Therefore, the aim of the present study was to examine the effect of pre-stroke treatment with A + D *vs.* A only on acute stroke severity and acute survival, as well as to examine the post-stroke effects of these drugs on all-cause mortality and cardiovascular morbidity. The study results are presented in the current paper.

## Methods

All acute ischaemic stroke/TIA patients within preceding 7days were consecutively recruited from the Stroke Unit, Department of Internal Medicine, Sahlgrenska University Hospital, from February 15 – May 31, 2009. No patients were included December 31, 2006 - September 3, 2007, due to data management. Patients with atrial fibrillation/flutter were excluded from the present analysis since use of A + D is not recommended in patients with atrial fibrillation. Stroke was defined according to World Health Organization definition [14]. TIA was defined as a reversible episode of neurologic deficit of ischaemic origin that resolved completely within 24 hours. In all patients a cerebral computed tomography (CT) scan was performed within 24 hours of admission.

For those patients with ischaemic stroke, not already on antiplatelet, treatment with antiplatelet was initiated after the initial CT scan. A detailed medical and drug history was obtained from each patient and next of kin from the attending physician with specific standardized questions regarding previous cardiovascular diseases. Medical records were thoroughly examined. Since the aim of the present study was to compare stroke outcome in patients with low-dose A *vs.* A + D, patients on other antiplatelet or anticoagulants were not included in this analysis.

On admission, the National Institute of Health Stroke Scale (NIHSS) [15,16] was used to assess stroke severity. All strokes were classified according to the TOAST criteria [17]. Systolic and diastolic blood pressures were measured in a supine position after 5 minutes rest at admittance, and on days 1, 2, 3, 7, and at discharge. Total cholesterol, triglycerides, high-density lipoprotein cholesterol (HDL-C) and low-density lipoprotein cholesterol (LDL-C) were measured by routine laboratory methods within the first 24 hours after admittance.

Cardiovascular end-points (coronary heart disease, congestive heart failure, and recurrent stroke/TIA) and medication were documented upon pre-scheduled 3 and 12-month follow-up outpatient clinic visits. NIHSS and Modified Rankin Scale (mRS) [18] were recorded at each visit. The medical records of these patients who did not show up at the scheduled visits was screened for cardiovascular endpoints. Recurrent stroke/TIA was defined as a stroke/TIA > 30days after the index stroke/TIA.

### Survival and functional outcome

Mortality data at 30 days, 3 and 12 months after index stroke/TIA was obtained from ELVIS (Swedish Population Registry). Data was, thus, extracted both from medical records and from interviews with patients in the ward during acute stroke and then at follow-up clinical visits. For those patients who did not attend the outpatient clinic visits, mRS was estimated by telephone interview with patients, next of kin or staff at nursing homes [19].

The study was approved by the Ethics Committee of The University of Göteborg. Written informed consent for participation in the study was obtained from the patients or their families. All patients were adults.

### Statistical analyses

The Mann–Whitney U test was used to compare the mean values of continuous variables (NIHSS, mRS) by treatment, while the chi-square test was used to compare categorical variables. Survival by treatment group was investigated by Cox proportional hazard regression where age, NIHSS at admission, as well as pre-stroke medical conditions (previous ischaemic stroke, previous myocardial infarction and type 2 diabetes) were used as covariates. Data are presented as hazard ratio (HR) with 95% confidence intervals (CI). P-values <0.05 were regarded as statistically significant (2-sided tests). All statistical analyses were performed by using SPSS, version 19.0. (Chicago, Illinois, USA).

## Results

Altogether 662 patients with ischaemic stroke (n = 554) or TIA (n = 108) were included in the study. Mean age was 76.8 ± 8.6 (Mean ± SD) years.

Baseline demographics and risk factors are summarized in Table 1.

**Table 1:** Baseline demography, risk factors and NIHSS in patients admitted for acute stroke/TIA (n = 662)

**Variables**	**% or Mean = ±SD**
Age(years)	77 ± 9
Male(%)	48.2
Female(%)	51.8
NIHSS at admittance	4 ± 6
(points)	
*Medical History*	
Previous stroke(%)	24.2
Myocardial	
infarction(%)	13.3
Congestive heart	
failure(%)	8.2
Hypertension(%)	53.9
Type 2 Diabetes	
Mellitus (%)	12.3

NIHSS: National Institute of Health Stroke Scale, SD: Standard Deviation.

Pre-stroke and post-stroke use of antithrombotic is shown in Table 2. The most frequent used doses were: 75mg aspirin daily and 200mg dipyridamole, twice daily.

**Table 2:** Use of antithrombotic pre-stroke medication and antithrombotic medication at discharge after acute stroke/TIA

**Drug**	**Pre-stroke use**	**Use at discharge**
	**(n = 662 patients)**	**(n = 628patients)**
Aspirin + Dipyridamole	62	275
Aspirin only	247	262
Clopidogrel	11	28
Warfarin	10	38
No use of antithrombotic	332	25

### Outcomes

Acute stroke severity, as measured by NIHSS, did not differ between patients on A + D , patients on A only, or patients with no antiplatelet treatment at all (not shown). The total 30-day mortality was 5.1% and no statistically significant difference was noted between the groups.

The cumulative all-cause mortality rates were 9.4% and 13.8%, at 3months and 12-months, respectively. The participation rate for 3 and 12-months follow-up clinic visits were 75% and 70%, respectively.

Altogether 628 patients with ischaemic stroke or TIA and without atrial fibrillation were discharged after acute stroke. At discharge there was no difference in systolic or diastolic blood pressure nor in total cholesterol, LDL-C, or triglycerides between users of A + D and users of aspirin only (not shown). HDL-C was slightly lower in the A + D group (1.46mmol/L ± 0.5; *vs*. 1.51mmol/L ± 0.42; p = 0.03).

### Survival

**Figure 1:**
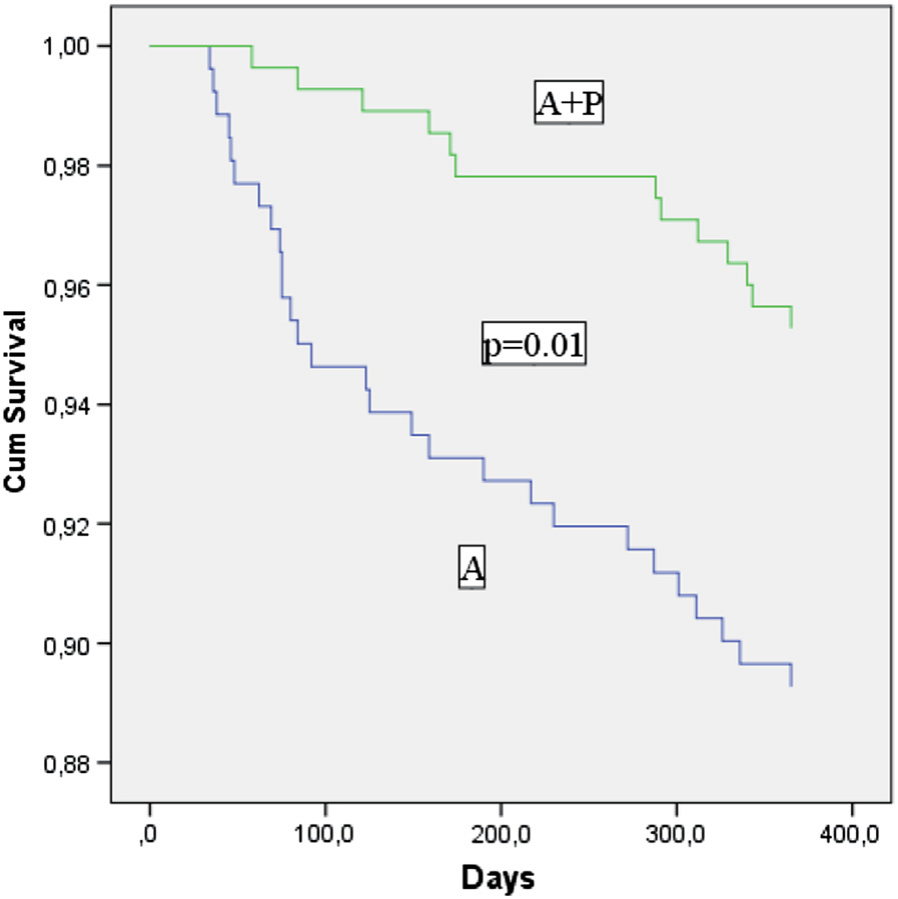
**Survival analysis.** Cumulative survival in patients treated with aspirin only or aspirin plus dipyridamole after initial acute ischaemic stroke/TIA. P-value for differences in 1-year survival between these two groups.

Survival analysis by Cox–proportional hazard model demonstrated that 12-months all-cause mortality was decreased in patients taking A + D compared with patients on aspirin only (HR, 0.52; CI, 032–0.86; p = 0.01) (Figure 1), after adjusting for age, NIHSS at admittance, previous ischaemic stroke, previous myocardial infarction and type 2 diabetes. Age (HR, 1.07; CI, 1.01-1.13; p = 0.021), and baseline NIHSS (HR, 1.08; CI, 1.02-1.14; p = 0.01), were also independent predictors of 12-months mortality.

There was also a tendency to decreased mortality, albeit not fully significant, in the A + D group in comparison with aspirin only group already after 3months (p = 0.12). There was, however, neither difference in functional outcome (mRS) at 3 and 12months, nor in occurrence rate of cardiovascular events (non-fatal myocardial infarction, stroke/TIA, ischemic heart disease or congestive heart failure) (Table 3).

**Table 3:** **Mortality at 3 and 12 months and cardiovascular events in patients using aspirin + dipyridamole (A + D)*vs.*use of aspirin only (A) after acute stroke/TIA (%)**

	**3-mo**	**12-mo**	**3-mo CVD**	**12-mo CVD**
Aspirin + Dipyridamole	1.1	5.7**	5.7	14.7
(n = 275)				
Aspirin only	5.7	10.7	3.8	10.9
(n = 262)				

** = P < 0.01 for comparison A + D *vs.*A only, : mortality, CVD: Cardiovascular Disease (recurring stroke/TIA, non-fatal myocardial infarction, congestive heart failure, ischemic heart disease), 3-mo: 3-months mortality; 12-mo: 12-months mortality. Results are presented in percent(%).

## Discussion

Our study shows that pre-stroke treatment with A + D does not reduce acute stroke severity in comparison with aspirin only. However, at the end of the 12-months follow-up period, patients on A + D had lower all-cause mortality compared with patients on A only. There was no difference in functional outcome and/or non-fatal cardiovascular events between patients on A + D or A only during follow-up.

In some previous studies [1-3], pre-stroke aspirin use has been associated with milder clinical deficits at stroke onset, but reduction in stroke severity may also differ by stroke mechanisms [20].

Few and, partly inconclusive, data are available on the effect of pre-stroke treatment with A + D on stroke severity [10]. Theoretically, D is suggested to be neuroprotective [7] and may reduce the inflammatory process. These effects in combination with the antiplatelet effects of aspirin might provide more efficient neuroprotection [10].

However, our study, in agreement with recent data [10] does not suggest that use of A + D before stroke onset would lessen severity of acute stroke. In spite of this, any conclusions must be drawn with caution since the number of patients using A + D before stroke was considerably smaller than the number of patients using aspirin only.

Our finding of reduced all-cause mortality in the A + D group at 12months follow-up is somewhat puzzling. Previously two large, randomised, studies [8,9] have shown A + D to be effective for secondary stroke prevention, but all-cause mortality was not reduced. Furthermore, in TIA patients The American-Canadian Co-Operative Study Group (21) has demonstrated that after a mean follow-up of 25 months there was no difference in mortality, stroke or retinal infarction between patients randomised to aspirin plus placebo or aspirin plus dipyridamole. There are several explanations for this discrepancy between our results and previous data. One explanation could be that the present study was non-randomised in contrast to previous randomised trials, which means that our results must be interpreted with extreme caution. In previous studies [8,9,21] the mean follow-up was between 24 months and 3.5 years in comparison with 12-months follow-up in the present study. During longer follow-up, risk factors for cardiovascular mortality may be more impending and may counteract the early positive effects of A + D.

The difference in mortality rate between A + D *vs.* aspirin only users in the present study was solid in spite of a smaller population sample and was demonstrated early after stroke. It may be hypothesized, that time from onset of stroke to initiation of antiplatelet therapy may be important.

In the present study acute stroke patients were enrolled if they had experienced a stroke/TIA within preceding 7 days. In previous, randomised studies [8,9,21] patients were eligible for inclusion in the non-acute setting i.e. the index stroke must have occurred within 3 and, respectively, 6 preceding months. Thus, in the present study treatment with antiplatelet was initiated very early and considering the suggested neuroprotective effects of dipyridamole and the antithrombotic properties of aspirin it may be hypothesized, that these early effects may be important for the significant reduction in all-cause mortality.

This association is further delineated by the clear difference in all-cause mortality between A + D *vs.* aspirin only users in the present study in comparison with previous studies [8,9,21].

Our patients were older, than in previous studies [8,9,21], which must be considered as a factor of potential importance. Increasing age may substantially augment mortality rate but in spite of this, the consistent effects on mortality persisted during the whole observational period. Furthermore, patients treated with Actilyse® (Boehringer-Ingelheim) were not included in the study. Only around 6.6% of all stroke patients were treated by Actilyse® during this period in Sweden so, tentatively, inclusion of Actilyse® treated patients would only have a marginal effect on the present results [22].

There was no difference at discharge in systolic or diastolic blood pressure nor in LDL-C, total cholesterol and triglycerides. If anything, HDL-C was slightly lower in the A + D group which, conversely, may suggest an increased risk profile in A + D users, but the difference was quite small. Therefore, it is not possible to discern a higher risk profile in the aspirin only group.

In disparity with previous studies [8,9] there was no difference in secondary stroke prevention in A + D users vs. users of aspirin only. Conceivably, this finding may depend on the low numbers of recurrent strokes during follow-up.

There are several limitations of the present work. This study was not randomised, but a post-hoc analysis of data collected from a stroke registry study. Our findings, which are in contradiction with results from several randomized trials, should therefore be interpreted with great caution. Our sample size was also much smaller than in previous randomised studies [8,9,21]. Numerous factors may influence use of A + D or aspirin only and these factors may be distributed dissimilarly among the two groups.

However, the influence of potential confounding factors such as age, stroke severity, previous stroke, previous myocardial infarction, and diabetes type 2, were taken into account and adjusted for in the survival analysis. We cannot, however, completely rule out that there are still some unknown factors that have substantially influenced our findings.

For pre-stroke use of A + D or aspirin only information was obtained from patients, next of kin or medical records, but there may be a bias even if information was meticulously collected. At discharge we recorded use of drugs but we cannot confirm that all patients remained on these drugs during follow-up.

The strength of the present study is that the population is large and that most patients have been followed for 12months.

Furthermore, all admitted stroke patients were included in the present study and there were very few patients lost to follow-up. However, our findings may not be valid for a general stroke population, but merely for a somewhat older stroke population, given the rather high mean age of our study population.

## Conclusions

In acute ischemic stroke/TIA pre-treatment with A + D does not seem to lessen clinical stroke severity. Nevertheless A + D seem to have beneficial effects on mortality up to one year after ischaemic stroke compared to aspirin only.

Based on these findings and considering the limitations mentioned above, we cautiously suggest that early initiation of these drugs in acute stroke may have an important protective effect and may improve survival, making the combination of A + D to be preferred to aspirin only. We must, however, as previously mentioned, be very cautious to draw any conclusion from the present data considering the non-randomised study design.

## Abbreviations

NIHSS: National Institute of Health Stroke Scale; mRS: Modified Rankin Scale; TOAST: Trial of Org 10172 in Acute Stroke Treatment.

## Competing interests

The authors declare that they have no competing interests.

## Authors’ contributions

LA worked with the database, performed calculations and assisted in writing the manuscript. CH worked with the database, performed statistical calculations and assisted in writing the manuscript. LB was one of the founders of the database and assisted in writing the manuscript. BA was the initiator of the present manuscript, performed statistical calculations and wrote a substantial part of the manuscript. All authors read and approved the final manuscript.

## Authors’ information

LA and CH are residents in Internal Medicine.

BA and LB are senior researchers in the field of clinical stroke research and both work as Senior Consultants at The Department of Internal Medicine, The Stroke Unit, at Sahlgrenska University Hospital, Göteborg, Sweden.

## Pre-publication history

The pre-publication history for this paper can be accessed here:

http://www.biomedcentral.com/1471-2377/12/67/prepub
